# Identification of the true hyperdiploid multiple myeloma subset by combining conventional karyotyping and FISH analysis

**DOI:** 10.1038/s41408-020-0285-6

**Published:** 2020-02-17

**Authors:** Gregorio Barilà, Laura Bonaldi, Angela Grassi, Annalisa Martines, Albana Liço, Nadia Macrì, Silvia Nalio, Laura Pavan, Tamara Berno, Antonio Branca, Giulia Calabretto, Marilena Carrino, Antonella Teramo, Sabrina Manni, Francesco Piazza, Gianpietro Semenzato, Renato Zambello

**Affiliations:** 1 Department of Medicine (DIMED), Hematology and Clinical Immunology section, Padua University School of Medicine, Padua, Italy; 2 Immunology and Molecular Oncology Unit, Veneto Institute of Oncology, IOV-IRCCS, Padua, Italy

**Keywords:** Myeloma, Myeloma

Multiple myeloma (MM) is a malignant disorder of plasma cells representing the second most common hematological malignancy^1^. The currently accepted MM pathogenetic model includes two different types of primary events, namely chromosome translocations or chromosome number alterations resulting in hyperdiploidy^2^. Primary translocations, often associated with hypodiploid karyotype, can be found in at least 40% of patients^3^. Among these translocations, t(4;14) and t(14;16), found in 10–15% and 2–3% of patients, respectively, are associated with a worse outcome, and included in the revised ISS^3,4^. More than half of MM cases, instead, have a hyperdiploid karyotype, characterized by trisomies involving odd chromosomes. In several studies, trisomic MM is associated with a favorable outcome^3,5^.

In addition to these primary events, secondary aberrations can be detected at diagnosis, or acquired following treatments, including gain of 1q (+1q) present in about 35–40% and associated with worse prognosis^6^, 13q deletion or monosomy 13 (del13q/−13), which were initially considered a poor prognostic marker only if present at the karyotype level^7^, and 17p deletion (17p−) found in 15–20% of MM and considered a high-risk disease marker, predictive of reduced survival^2–4,6,8^.

Cytogenetic abnormalities in MM can be studied by conventional karyotyping (CK) or fluorescent in situ hybridization (FISH) analysis, and more recently by array-comparative genomic hybridization (Array-CGH) and single-nucleotide polymorphism array (SNP-array). Although karyotyping may ideally describe all chromosome aberrations of the neoplastic clone, with the exception of some cryptic translocations, the amount of proliferating cells that are required hampered the routine use of CK. For this reason, in the latest European Myeloma Network (EMN) guidelines, FISH analysis on plasma cells is recommended, and should include at least t(4;14) and 17p− abnormalities, with t(14;16) and +1q suggested as well^9^. Hyperdiploid MM (HRD MM), instead, is not routinely investigated because multiple probes are required over the conventional markers. However, in the era of novel agents, the identification of hyperdiploidy was proved to be helpful in MM prognostication since trisomic MM can benefit the most from lenalidomide treatment, and retains a favorable outcome^3,5^.

Starting from CK, our study aims at identifying distinctive genetic features of hyperdiploid MM that are associated with a favorable outcome, pursuing the goal of their inclusion in the routine FISH assessment with a step-by-step approach.

Bone marrow of 292 newly diagnosed MM patients was studied by both CK and FISH on separated plasma cells for detecting high-risk (HR) cytogenetic aberrations, including t(4;14), t(14;16), 17p−, and +1q. Patient’s characteristics, including ISS stage, symptoms at diagnosis, type of treatment, and OS, were collected (Supplementary). Median follow-up of the study population was 42 months.

Among the entire cohort, 76 (26%) patients showed an abnormal karyotype, and were selected for further analysis, while the remnant 216 cases were not evaluable or not informative. Based on CK nomenclature, karyotypes were classified into hyperdiploid (HRD, 47–57 chromosomes) and hypodiploid (HD, 35–45 chromosomes), leading to two groups of patients: 50 HRD MM (66%) and 26 HD MM patients (34%). The clinical and biological features of the two groups are reported in Supplementary Table 1. All patients received treatment with novel agents, including bortezomib (74/76, 97%), lenalidomide (52/76, 68%), or both (51/76, 67%), and 14 patients (14/76, 18%) were treated with pomalidomide. Moreover, 29 (38%) patients received autologous stem cell transplantation (ASCT). No significant differences were found in age, sex, stage, and clinical presentation between the two subsets of patients, although a trend to a more aggressive disease characterized HD MM, with a higher frequency of ISS III (57.7% vs. 38.8%), higher LDH levels (32% vs. 12.8%), and higher frequency of renal injury (30.8% vs. 14.0%) and hypercalcemia at diagnosis (23.1% vs. 6%). Concerning cytogenetics, HD MM patients were characterized by a significantly higher frequency of HR FISH alterations [t(4;14), t(14;16), and 17p− (63.2% vs. 20.5%, *p* = 0.0028)], of del13q/−13 by CK (64.0% vs. 22.4%, *p* = 0.0008), and immunoglobulin heavy locus (*IGH*) translocation (66.7% vs. 29.2%, *p* = 0.0053), but no difference in +1q frequency was found (36.0% vs. 42.9%, *p* = 0.624). Despite the fact that hypodiploidy is a recognized adverse prognostic factor^10^, in our cohort OS was not significantly different between HD MM and HRD MM patients (42 vs. 53 months, *p* = 0.5, Supplementary Fig. 1). This unexpected result led us to focus on the cytogenetic features of HRD MM patients.

Overall, within HRD MM cases, trisomies of chromosomes 3 (31/49, 63%), 5 (29/49, 59%), 9 (37/48, 77%), 11 (34/49, 69%), 15 (27/49, 55%), and 19 (31/49, 63%) were the most represented. HR chromosomal changes were also detected in 30/50 (57.7%) cases, including 21/49 (42.9%) with +1q, 1/44 (2%) with t(4;14), and 8/45 (18%) with 17p−. In addition, in 14/48 patients (29.2%), a *IGH* rearrangement was detected by both karyotype and FISH analysis (Supplementary Table 1).

In this subset, the major features associated with decreased OS were ≥2 alterations by FISH (32 vs. 57 months, *p* = 0.0123), *IGH* rearrangement (32 vs. 57 months, *p* = 0.0319), and +1q (39 vs. 56 months, weakly significant with *p* = 0.0929). On the contrary, features associated with a better outcome were co-occurrence of trisomy of 9/11/15 chromosomes (62 vs. 39 months, *p* = 0.0218) and ASCT (80 vs. 43 months, *p* = 0.0465) (Fig. 1a–e). According to these results, we classified HRD MM based on the number of trisomies into trisomic HRD MM (T-HRD MM, *n* = 26) with ≥5 trisomies and a non-trisomic group (N-HRD MM, *n* = 16), with <5 trisomies.

**Fig. 1:**
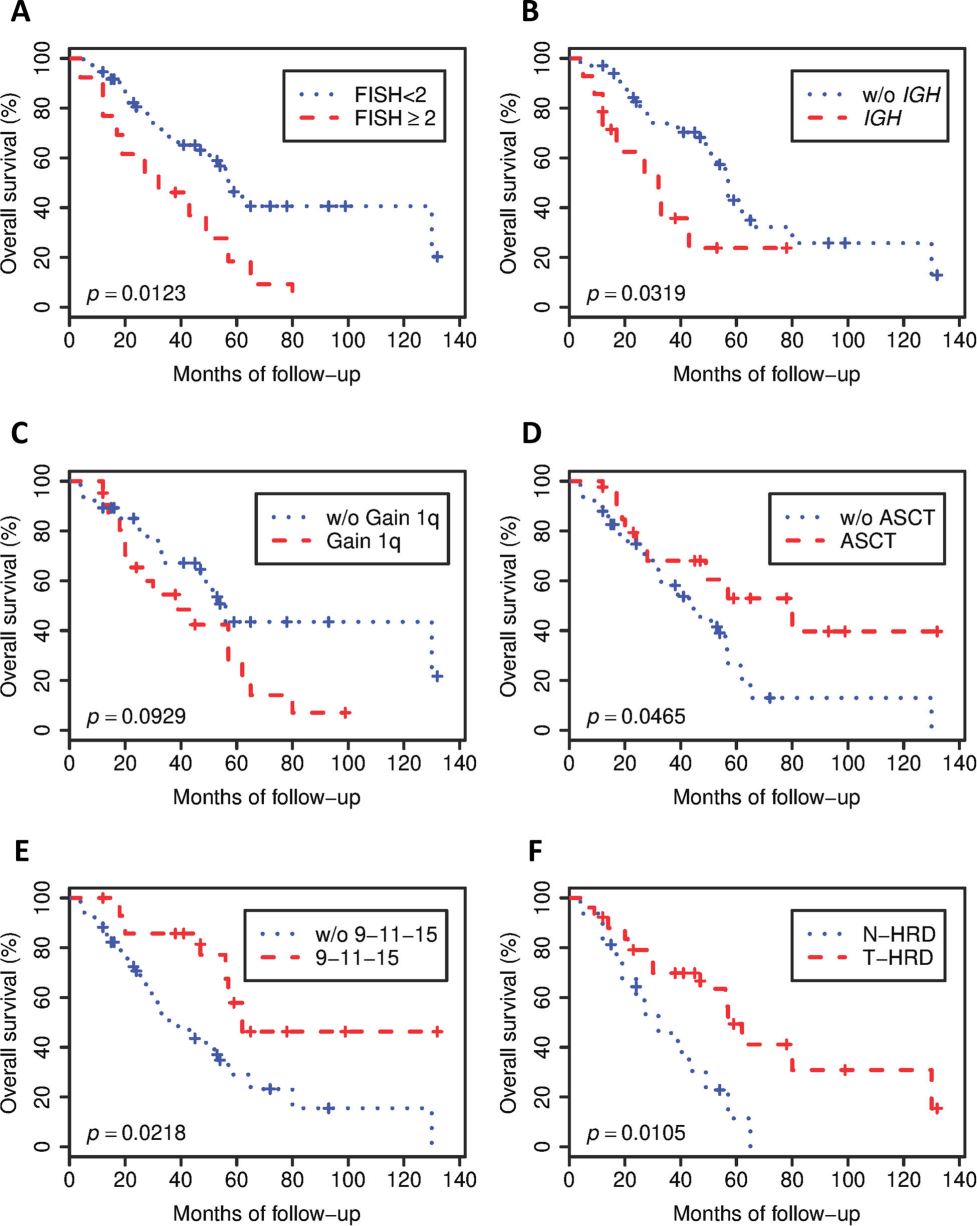
Kaplan–Meier curves showing overall survival of HRD MM patients according to cytogenetic and FISH features. **a** According to the number of FISH alterations (≥2 or <2) (*p* = 0.0123). **b** Based on the presence or absence of *IGH* rearrangement (*p* = 0.0319). **c** According to the presence of 1q gain (*p* = 0.0929). **d** Depending on the transplant status (*p* = 0.0465). **e** According to the concomitant presence of 9/11/15 trisomies (*p* = 0.0218). **f** Based on T-HRD MM (≥5 trisomies) and N-HRD MM (<5 trisomies) classification (*p* = 0.0105). The comparisons between groups were made by log-rank test. HRD MM hyperdiploid multiple myeloma, FISH fluorescent in situ hybridization, *IGH* immunoglobulin heavy locus, ASCT autologous stem cell transplantation, N-HRD MM non-trisomic hyperdiploid multiple myeloma, T-HRD MM trisomic hyperdiploid multiple myeloma.

Interestingly, T-HRD MM patients were characterized by a better outcome than N-HRD MM (57 vs. 32 months, *p* = 0.0105, Fig. 1f). Moreover, T-HRD MM was associated with lower rates of FISH alterations (≥2 alterations, 7.7% vs. 68.7%, *p* = 0.0001) and HR FISH aberrations (4.8% vs. 50%, *p* = 0.0023). Although not supported by a statistical significance, both del13q/−13 (15.4% vs. 37.5%, *p* = 0.1422) and *IGH* translocations (20.0% vs. 43.8%, *p* = 0.1606) were less represented in T-HRD MM. Overall, these observations support the hypothesis that T-HRD MM accounts for the classical definition of HRD MM, and more specifically that concurrent trisomy of 9/11/15 represents a surrogate marker of true HRD MM, being present in 53.8% of T-HRD MM patients and completely absent in N-HRD MM (*p* = 0.0002, Table 1).

**Table 1:** Biological features of T-HRD MM and N-HRD MM.

	T-HRD (*n* = 26)	N-HRD (*n* = 16)	*p* Value^*^
*IGH* translocation	5/25 (20%)	7/16 (43.8%)	0.1606
High-risk FISH^**^	1/21 (4.8%)	8/16 (50%)	**0.0023**
Gain 1q	11/26 (42.3%)	9/16 (56.2%)	0.5265
Del13q/−13	4/26 (15.4%)	6/16 (37.5%)	0.1422
≥2 FISH alterations	2/26 (7.7%)	11/16 (68.7%)	**0.0001**
9–11–15 trisomy	14/26 (53.8%)	0/16 (0%)	**0.0002**

*p Values are calculated using Fisher’s exact test.

**Includes t(4;14), t(14;16), and 17p−.

*MM* multiple myeloma, *HRD* hyperdiploid, *T-HRD* trisomic hyperdiploid, *N-HRD* non-trisomic hyperdiploid, *IGH* immunoglobulin heavy locus, *FISH* fluorescent in situ hybridization.

By univariate analysis, *IGH* translocations, concomitant trisomies of 9/11/15, ≥2 FISH alterations, and stratification in T-HRD and N-HRD were the most important prognostic factors, while ASCT and +1q were only weakly significant (Supplementary Table 2). In multivariate analysis, concomitant trisomies 9/11/15 was the most powerful prognostic factor (*p* < 0.01, HR = 0.28; 95% CI: 0.10–0.74), followed by *IGH* rearrangement (*p* < 0.04, HR = 2.42; 95% CI: 1.04–5.64), although in the opposite direction (Supplementary Table 3).

In our cohort, HRD MM OS was not significantly different with respect to HD MM, despite an increased presence of HR genetic and clinical features in HD MM patients. This can be only partially related to the not negligible presence in HRD MM patients of HR alterations, like those detected by FISH. It is also possible that a cytogenetic clone unintentionally selects a more aggressive form of MM, therefore making the difference elusive between the two groups^11,12^. In fact, the possibility of identifying an abnormal clone by CK has been related to a high mitotic rate and high percentage of bone marrow plasma cells, these variables also being correlated with the percentage of abnormal metaphases. Alternatively, it is also possible that the development of chromosome abnormalities in the malignant plasma cell may lead to a more aggressive tumor cell growth. Despite the limitations of the study (retrospective nature, small sample size, and heterogeneous treatments received), in our cohort, it clearly appears that cytogenetically defined HRD MM represents a heterogeneous group of MM where numerical changes are coupled with structural aberrations. The same evidence was reported by other groups that demonstrated that trisomies and high-risk cytogenetic alterations could coexist^5,13,14^, although with conflicting results on the outcome. Consequently, it is evident that the number of chromosomes or the ploidy level could not be enough to define the HRD MM, but rather the whole pattern of chromosome aberrations is needed to identify myeloma with hyperdiploidy. Indeed, in our cohort, we confirmed that the type and number of trisomies detected by CK have a relevance since concomitant 9/11/15 trisomies correlate to a better outcome, and represent an independent prognostic factor together with the absence of *IGH* rearrangement. Moreover, this association was particularly evident in T-HRD patients, where trisomies correlate with low frequencies (≤2) of FISH alterations and HR FISH.

Although at least half of MM patients belong to the HRD subset, the latest EMN guidelines do not recommend the assessment of hyperdiploid status at diagnosis^9^. The major issue of evaluating hyperdiploidy by FISH is the need of multiple probes for odd chromosomes that increases the costs, the effort of testing, and the amount of plasma cells required. Indeed, our results suggest to restrict the investigation of hyperdiploidy to those cases that in the beginning are negative for HR FISH, and simultaneously have ≤2 FISH abnormalities and no *IGH* rearrangement. This approach would save resources, and at the same time, would make the characterization of most of not high-risk samples possible.

The detection of ploidy status is an emerging issue, especially for HRD MM, and in a recent paper, Sidana et al. developed a flow cytometry approach based on the DNA index. With this method, high-hyperdiploidy patients had an improved OS as compared with those with low-hyperdiploidy^15^, consistent with our CK-based results.

In conclusion, the identification of T-HRD MM represents a new challenge within the heterogeneous group of HRD patients, with relevant prognostic implications. The combination of banding analysis and FISH for HR aberrations contributes to better define the complexity of HRD MM. Finally, the concomitant presence of trisomies of chromosomes 9/11/15, after exclusion of HR features, is a surrogate marker of true hyperdiploid myeloma.

## Supplementary information

Supplementary Material

## References

[1] Palumbo A, Anderson K (2011). Multiple myeloma. N. Engl. J. Med..

[2] Morgan GJ, Walker BA, Davies FE (2012). The genetic architecture of multiple myeloma. Nat. Rev. Cancer.

[3] Kumar SK, Rajkumar SV (2018). The multiple myelomas—current concepts in cytogenetic classification and therapy. Nat. Rev. Clin. Oncol..

[4] Manier S (2017). Genomic complexity of multiple myeloma and its clinical implications. Nat. Rev. Clin. Oncol..

[5] Kumar S (2012). Trisomies in multiple myeloma: impact on survival in patients with high-risk cytogenetics. Blood.

[6] Avet-Loiseau H (2012). Long-term analysis of the IFM 99 trials for myeloma: cytogenetic abnormalities [t(4;14), del(17p), 1q gains] play a major role in defining long-term survival. J. Clin. Oncol..

[7] Chiecchio L (2006). Deletion of chromosome 13 detected by conventional cytogenetics is a critical prognostic factor in myeloma. Leukemia.

[8] Sonneveld P (2016). Treatment of multiple myeloma with high-risk cytogenetics: a consensus of the International Myeloma Working Group. Blood.

[9] Caers J (2018). European Myeloma Network recommendations on tools for the diagnosis and monitoring of multiple myeloma: what to use and when. Haematologica.

[10] Van Wier S (2013). Hypodiploid multiple myeloma is characterized by more aggressive molecular markers than non-hyperdiploid multiple myeloma. Haematologica.

[11] Rajan AM, Rajkumar SV (2015). Interpretation of cytogenetic results in multiple myeloma for clinical practice. Blood Cancer J..

[12] Fonseca R (2004). Genetics and cytogenetics of multiple myeloma: a workshop report. Cancer Res..

[13] Pawlyn C (2015). Coexistent hyperdiploidy does not abrogate poor prognosis in myeloma with adverse cytogenetics and may precede IGH translocations. Blood.

[14] Chretien ML (2015). Understanding the role of hyperdiploidy in myeloma prognosis: which trisomies really matter?. Blood.

[15] Sidana S (2019). Rapid assessment of hyperdiploidy in plasma cell disorders using a novel multi-parametric flow cytometry method. Am. J. Hematol..

