# Supplementary material for: Identification of the true hyperdiploid multiple myeloma subset by combining conventional karyotyping and FISH analysis
Source: Blood Cancer J. 2020 Feb 17;10(2):18. doi: 10.1038/s41408-020-0285-6 (PMC7026173; doi:10.1038/s41408-020-0285-6)
Supplement: Supplementary file 1 — Supplementary Material [file 41408_2020_285_MOESM1_ESM.pdf]

Supplementary Table 1 - Clinical and biological features of MM patients' cohort

	HRD (n=50)	HD (n=26)	p-value*
**Mean Age (SE)**	66.36 (1.58)	67.46 (2.40)	p=0.6942
**Sex Male**	29/50 (58.0%)	12/26 (46.2%)	p=0.3442
**ISS III**	19/50 (38.0%)	15/26 (57.7%)	p=0.1447
**DS III**	35/50 (70.0%)	17/26 (65.4%)	p=0.7958
**PC>60%**	30/50 (60.0%)	18/26 (69.2%)	p=0.4641
**High LDH**	6/47 (12.8%)	8/25 (32.0%)	p=0.0645
**Hypercalcemia**	3/50 (6.0%)	6/26 (23.1%)	p=0.0555
**Renal Injury**	7/50 (14.0%)	8/26 (30.8%)	p=0.1270
**Anemia**	40/50 (80.0%)	25/26 (96.2%)	p=0.0857
**Lytic bone lesion**	34/50 (68.0%)	17/26 (65.4%)	p=1.0000
**ASCT**	21/50 (42.0%)	8/26 (30.8%)	p=0.4564
**High risk FISH****	9/44 (20.5%)	12/19 (63.2%)	**p=0.0028**
**del13q/-13**	11/49 (22.4%)	16/25 (64.0%)	**p=0.0008**
**Gain 1q**	21/49 (42.9%)	9/25 (36.0%)	p=0.6239
***IGH* translocation**	14/48 (29.2%)	16/24 (66.7%)	**p=0.0053**

*P-values for count data were calculated using Fisher's exact test. For the variable Age a two sample t-test was applied.
**Including t(4;14), t(14;16) and 17p-

Abbreviations. MM: Multiple Myeloma, HRD: hyperdiploid, HD: hypodiploid, ISS: international staging system, DS: Durie Salmon, PC: plasmacells, ASCT: autologous stem cell transplantation, FISH: fluorescent *in situ* hybridization, *IGH*: immunoglobulin heavy locus

Supplementary Table 2 - Univariate Cox regression analysis in HRD MM patients

**Covariate**	**HR (95% CI)**	**p-value**
**Age>65**	1.69 (0.79-3.62)	**0.1780**
**Sex male**	1.30 (0.60-2.80)	0.5010
**ISS III**	1.61 (0.76-3.38)	0.2130
**ASCT**	0.45 (0.20-1.00)	**0.0523**
**High Risk FISH**	1.57 (0.68-3.66)	0.2930
**FISH≥2**	2.53 (1.20-5.36)	**0.0152**
***IGH* translocation**	2.41 (1.05-5.51)	**0.0373**
**Gain 1q**	1.90 (0.89-4.02)	**0.0954**
**9-11-15**	0.36 (0.15-0.90)	**0.0280**
**T-HRD**	0.36 (0.16-0.81)	**0.0134**

Table showing the clinical and biological variables evaluated by univariate Cox regression modelling. The variables yielding a p-value <0.2 (in bold) were selected to perform also a multivariate Cox regression analysis.

Abbreviations. HRD: hyperdiploid; MM: Multiple Myeloma; HR: hazard ratio; ISS: international staging system; ASCT: autologous stem cell transplantation; FISH: fluorescent *in situ* hybridization; *IGH*: immunoglobulin heavy locus, T-HRD: trisomic hyperdiploid.

Supplementary Table 3 – Multivariate Cox regression analysis in HRD MM patients

**Covariate**	**HR (95% CI)**	**p-value**
***IGH* translocation**	2.42 (1.04-5.64)	**0.0411**
**9-11-15**	0.28 (0.10-0.74)	**0.0105**

A backward stepwise selection procedure was applied to retain only the significant factors in the multivariate Cox regression model. The Table shows the final model, in which only two biological variables, i.e. concomitant presence of 9/11/15 trisomies (p=0.0105) and *IGH* translocation (p=0.0411), resulted significant.

Abbreviations. HRD: hyperdiploid, MM: Multiple Myeloma, *IGH*: immunoglobulin heavy locus, HR: hazard ratio.

Supplementary Figure 1- Overall Survival of HRD and HD Multiple Myeloma patients

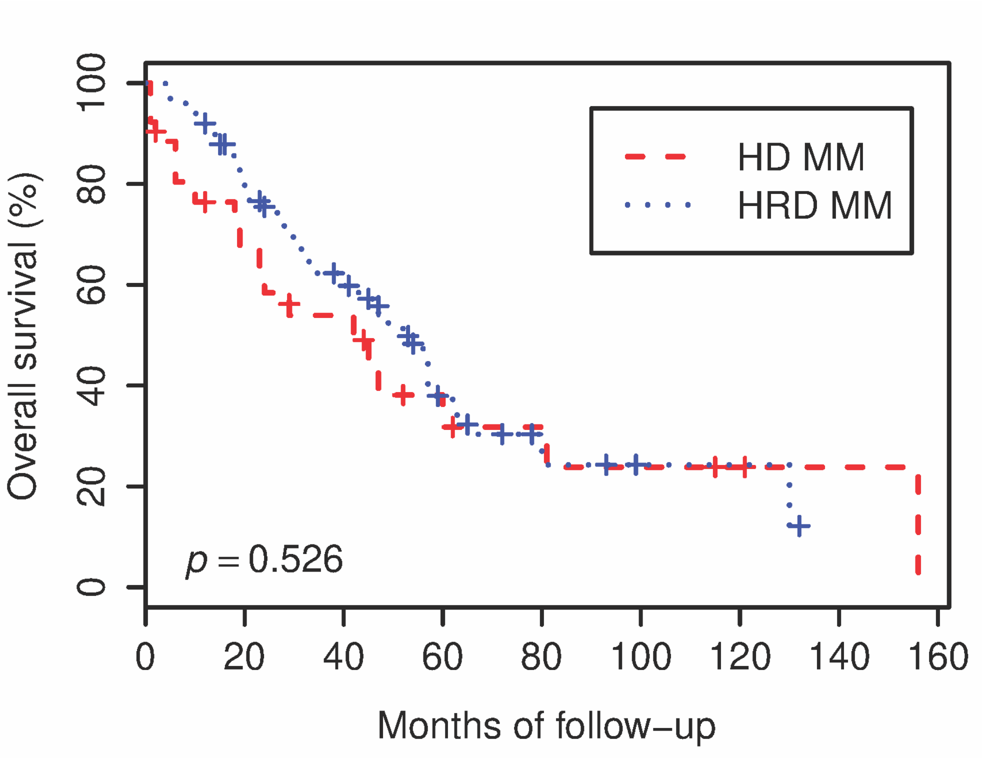

Kaplan-Meier curves showing overall survival of HRD MM and HD MM patients. The comparison between the two groups was made using log-rank test.

Abbreviations. HRD MM: hyperdiploid Multiple Myeloma, HD MM: hypodiploid Multiple Myeloma.

SUPPLEMENTARY METHODS

Study population

A cohort of 292 patients affected by Multiple Myeloma and followed by the Hematology Unit of Padua University Hospital was included in this study.

This study was performed according to the Helsinki Declaration and patients gave written informed consensus prior to inclusion in the study. The protocol (PD-MM-REG1) and informed consent form were approved by Padua ethical committee.

Statistical Analysis

The Fisher's exact test was used to compare the features of interest between HRD MM and HD MM and between T-HRD and N-HRD.

Overall survival (OS) was defined as the time from MM diagnosis until death due to any cause. Patients were censored at the time of last follow-up if no event was reported. Survival time curves and median OS were obtained by the Kaplan-Meier method, and comparisons between groups were made by log-rank test. Association between single factors (either clinical or cytogenetic) and OS was evaluated using a univariate Cox model. The variables yielding a p-value<0.2 in univariate Cox regression (i.e. age>65, transplantation status, FISH≥2, *IGH* translocation, gain1q, 9-11-15 trisomy, T-HRD status, Supplementary Table 2) were selected to perform a multivariate Cox regression analysis. A backward stepwise selection procedure was then applied to identify, among clinical and biological variables, the significant factors retained in the final multivariate model. Hazard ratios (HR) and 95% confidence intervals were calculated for both univariate and multivariate models.

Statistical analysis was performed in the R environment (v. 3.5.1), using the 'survival' package and custom code.

Conventional karyotyping analysis

Chromosome banding analysis was performed on bone marrow samples after short term culture (24 and/or 48 hours) in RPMI 1640 medium (Euroclone, Pero (MI), Italy) supplemented with 20% of fetal calf serum (Life technologies, Carlsbad, California, US). Slides were prepared by conventional methods and G-banding was obtained by Wright's stain (Sigma Aldrich Co., St Louis, Missouri, US).

At least 25 metaphases were analysed for each sample and karyotype was described according to the International System for Human Cytogenetic Nomenclature (ISCN 2016)[1].

Interphase cytogenetic analysis

FISH analysis was carried out on separated plasma cells obtained after positive selection with CD138+ microbeads using Automacs Proseparator (Miltenyi, Bergisch Gladbach, Germany) with locus specific probes for high risk abnormalities. Probes for t(4;14)(p16;q32) and t(14;16)(q32;q23) translocations (LSI FGFR3/IGH, LSI IGH/MAF Abbott, Chicago, Illinois, US), for deletion of short arm of chromosome 17 at TP53 locus 17p13.1 (LSI TP53/CEP17, Abbott) and for amplification/gain of locus 1q21 (CKS1B) (XL CDKN2C/CKS1B MetaSystems, Altlussheim, Germany) were used according to manufacturer's protocol. One hundred interphase nuclei were analysed for each probe with AxioImager Z2 microscope (Zeiss, Jena, Germany) equipped with appropriate filters. The cut-off for positive values were: 10% for 17p13 deletion and gain/amplification or 1q21 and 5% for translocations with dual fusion probes.

Supplementary reference

1. McGowan-Jordan J. SA, Schmid M. *An International System for Human Cytogenomic Nomenclature (2016)*, 1st edn. Karger: New York, 2016.
